# Role of the Dietary Phytochemical Curcumin in Targeting Cancer Cell Signalling Pathways

**DOI:** 10.3390/plants12091782

**Published:** 2023-04-26

**Authors:** Abhay Prakash Mishra, Swetanshu, Pratichi Singh, Shikha Yadav, Manisha Nigam, Veronique Seidel, Celia Fortuna Rodrigues

**Affiliations:** 1 Department of Pharmacology, Faculty of Health Science, University of Free State, Bloemfontein 9300, South Africa; 2 Department of Biosciences, School of Basic and Applied Sciences, Galgotias University, Greater Noida 203201, Uttar Pradesh, India; 3 Department of Pharmacy, School of Medical and Allied Sciences, Galgotias University, Greater Noida 203201, Uttar Pradesh, India; 4 Department of Biochemistry, H. N. B. Garhwal University, Srinagar Garhwal 246174, Uttarakhand, India; 5 Natural Products Research Laboratory, Strathclyde Institute of Pharmacy and Biomedical Sciences, University of Strathclyde, 161 Cathedral Street, Glasgow G4 0RE, UK; 6 LEPABE—Laboratory for Process Engineering, Environment, Biotechnology and Energy, Faculty of Engineering, University of Porto, Rua Dr. Roberto Frias, 4200-465 Porto, Portugal; 7 ALiCE—Associate Laboratory in Chemical Engineering, Faculty of Engineering, University of Porto, Rua Dr. Roberto Frias, 4200-465 Porto, Portugal; 8 TOXRUN—Toxicology Research Unit, Cooperativa de Ensino Superior Politécnico e Universitário—CESPU, 4585-116 Gandra PRD, Portugal

**Keywords:** cancer, curcumin, signalling pathways

## Abstract

The diarylheptanoid curcumin [(1*E*,6*E*)-1,7-bis(4-hydroxy-3-methoxyphenyl)hepta-1,6-diene-3,5-dione] is one of the phenolic pigments responsible for the yellow colour of turmeric (*Curcuma longa* L.). This phytochemical has gained much attention in recent years due to its therapeutic potential in cancer. A range of drug delivery approaches have been developed to optimise the pharmacokinetic profile of curcumin and ensure that it reaches its target sites. Curcumin exhibits numerous biological effects, including anti-inflammatory, cardioprotective, antidiabetic, and anti-aging activities. It has also been extensively studied for its role as a cancer chemopreventive and anticancer agent. This review focusses on the role of curcumin in targeting the cell signalling pathways involved in cancer, particularly via modulation of growth factors, transcription factors, kinases and other enzymes, pro-inflammatory cytokines, and pro-apoptotic and anti-apoptotic proteins. It is hoped that this study will help future work on the potential of curcumin to fight cancer.

## 1. Introduction

**Figure 1:**
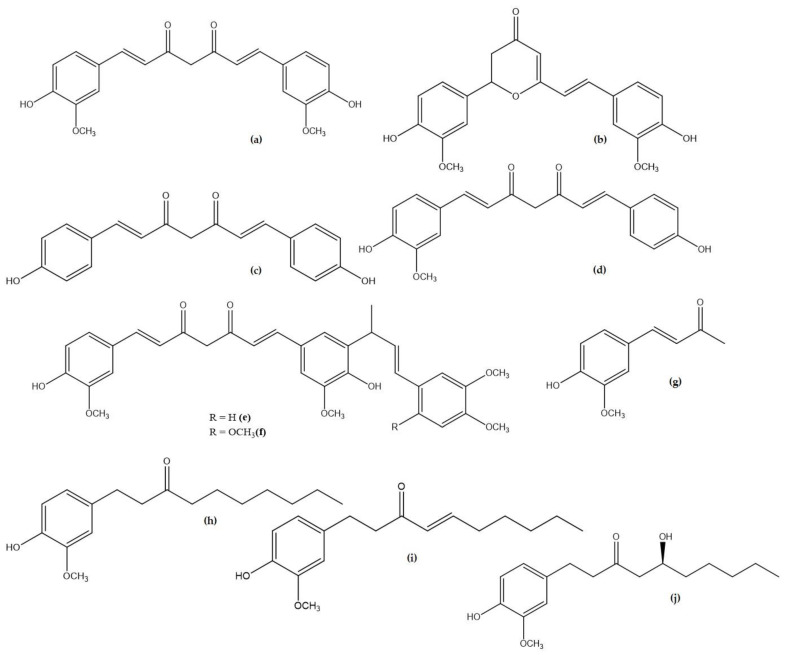
Chemical structures of curcumin (**a**) and other curcuminoids (**b**–**j**).

Curcumin (C_21_H_20_O_6_), also known as [(1*E*,6*E*)-1,7-bis(4-hydroxy-3-methoxyphenyl)hepta-1,6-diene-3,5-dione] or diferuloylmethane, is a crystalline substance with a bright orange-yellow colour that is used as a dye and food colouring agent, mainly in the Indian subcontinent. It is most commonly found, along with related compounds collectively known as the curcuminoids, in the rhizome of the spice turmeric (*Curcuma longa* L.) as well as in other plants from the Zingiberaceae family (Table 1, Figure 1). The amount of curcumin in food such as turmeric is influenced by environmental factors such as climate, soil type, and methods used to process the plant material. The content of curcuminoids in *C. longa* has been estimated to range between 1–2 µg/g [1]. 

Curcumin has a moderate to low degree of solubility in water and a low bioavailability [1,2] (Table 2). When consumed orally, it is moderately absorbed via the gastrointestinal tract and gets rapidly metabolized in the liver, small intestine and kidney, mostly by reduction and conjugation as curcumin sulphate, curcumin glucuronide and methylated curcumin. Thereafter, it is excreted out via the faeces and urine. Studies have reported that curcumin metabolites, among which tetra and hexahydrocurcumin and tetrahydrocurcumin are the most predominant, contribute to the various pharmacological properties of curcumin [3,4,5]. The gender of an individual can affect the pharmacokinetics of curcumin. Studies have revealed that females show 1.4 to 2.1 times higher levels of curcuminoids in their plasma than males after oral administration [6,7]. A significant level research has been carried out attempting to increase the bioavailability of curcumin, including using nanoparticles, liposomes, polymeric micelles, phospholipid complexes, and administering curcumin in combination with other substances such as piperine (Table 3).

Curcumin has various health benefits, including anti-inflammatory, anti-allergic, antioxidant, and anticancer properties [8]. In India, around 1.4 million people are diagnosed with cancer each year, causing 1.2 million deaths annually. In 2020, it was estimated that around 10 million people died due to cancer worldwide. The majority of cases included deaths from lung cancer in males and from breast or cervical cancer in females [9]. 

The purpose of this review is to discuss the role of curcumin in cancer, with a particular focus on the cell-signalling pathways targeted by curcumin. Laboratory studies carried out to date on animal models suggest that curcumin might have therapeutic potential in cancer. Although these studies are still in the early stage, curcumin remains a promising phytochemical to consider in cancer discovery and development given its significant role in numerous cancer-cell signalling pathways.

## 2. Methodology

Search engines including Google Scholar, PubMed and Medline were used to retrieve the relevant literature. Almost 200 articles, including original research, review papers, and book chapters, all published between 2000 and 2022, were used to gather relevant information. The primary search terms were ‘curcumin and clinical studies’, ‘curcumin and bioavailability’, ‘curcumin and breast cancer’, ‘curcumin and prostate cancer’, ‘curcumin and brain cancer’, ‘curcumin and pancreatic cancer’, ‘curcumin and gastric cancer’, ‘curcumin and leukaemia’, and ‘curcumin and nutraceuticals’. Each article was carefully read, and it was ensured that no information was duplicated. ACD/ChemSketch (2021.2.1) was used to draw all chemical structures.

## 3. Curcumin and Cancer: In Vitro and In Vivo Studies

Curcumin, either alone or in combination with other anticancer drugs, is able to modulate various molecular targets and signalling pathways involved in cancer (Table 4). The sections below discuss the effects of curcumin on various types of cancer, namely lung, breast, prostate, brain, pancreatic, gastric and leukaemia.

### 3.1. Lung Cancer 

Lung cancer is mostly prevalent in males rather than females [10]. Common treatments for lung cancer involve chemotherapy, radiation therapy, immunotherapy and surgery [11]. Curcumin has been shown to modulate the wingless/integrated Wnt/β-catenin pathway in A549 lung cancer cells. It downregulates the expression of the nuclear factor-κB (NF-κB) and of the vascular endothelial growth factor (VEGF) in that cell line [12]. It also inhibits the expression of the enhancer of zeste homolog 2 (EZH2) in cancerous cells, which eventually downregulates the expression of the gene coding for the neurogenic locus notch homolog protein 1 (Notch 1) [13]. Curcumin has been reported to stop cell division at the G2/M phase, increase cell apoptosis, and show an antiproliferative effect on non-small-cell lung cancer (NSCLC) cells via activating reactive oxidative species (ROS)-DNA damage [14]. The ROS-mediated apoptosis and migration-blocking of lung cancer cells was also reported for a curcumin synthetic derivative [15]. Curcumin has also been shown to inhibit the phosphoinositide 3-kinase (PI3K)/Akt-dependent pathway, leading to apoptosis in various lung cancer cells [16]. This was also observed when administered combined with Paris saponin II (a chemical extracted from the rhizomes of *Paris polyphilla*) [17]. In addition, curcumin enhanced the effects of the cancer chemotherapeutics cisplatin and gefitinib, increasing their antiproliferative ability and inducing apoptosis [18,19]. 

### 3.2. Breast Cancer 

Breast cancer is the most common type of cancer in women worldwide. Modern treatment approaches involve targeting the production of molecules such as NF-κB, the human epidermal growth factor receptor 2 (Her-2), Notch, and signal transducer and activator of transcription 3 (STAT-3) [20,21,22]. The Akt/mTOR-dependent pathway is a predominant signalling pathway associated with breast cancer, and many clinical trials have confirmed that targeting this pathway could lead to promising therapeutic activity [23]. Curcumin has been reported to interfere with the phosphorylation of Akt and the mechanistic target of rapamycin (mTOR) in MCF7 and T47D breast cancer cells [24]. The activation of NF-κB also plays an important role in cancer and has been linked with the invasion, proliferation, and metastasis of breast cancer cells. Curcumin can inhibit the nuclear translocation of NF-κB, reducing the levels of p100 and p52 in MCF-7 and MDA-MB-453 breast cancer cells [24]. Its cytotoxicity on MCF-7 cells has been linked with the enhanced expression of the spermidine/spermine N1-acetyltransferase (SSAT) gene, which is also associated with the NF-κB-dependent signalling pathway [25]. Curcumin has also been reported to inactivate the autocrine growth hormone (GH) signalling pathway in T47D cancer cells as well as reduce the release of anti-apoptotic proteins Bcl-2 and Bcl-xl [26]. Curcumin reduces the overexpression of flap endonuclease 1 (FEN1), an enzyme associated with cisplatin-resistance in breast cancer cells, thereby increasing the sensitivity of cancer cells to this chemotherapeutic agent [27]. Finally, curcumin also downregulates the expression of the multidrug resistance mutation 1 (MDR-1) gene in paclitaxel-resistant cells [28]. 

### 3.3. Prostate Cancer

In the western world, prostate cancer ranks second in the types of cancers affecting men [29]. One approach to treat this type of cancer is the use of drugs that inhibit the androgen receptor (AR)-dependent signalling pathway [30,31]. In studies carried out on prostate cancer cells, curcumin has been reported to interact with the mitogen-activated protein kinase (MAPK), epidermal growth factor receptor (EGFR), and NF-κB signalling pathways [32]. It can inactivate NF-κB, suppressing the release of inflammatory mediators such as interleukin (IL)-6. It is also able to reduce the levels of cyclooxygenase (COX)-2, Bcl-2, and Bcl-xL [33,34]. In androgen-independent (AI) PC-3 prostate cancer cells, curcumin has been reported to inactivate the NF-κB pathway and suppress the C-X-C motif chemokine ligand 1 (CXCL-1) and CXCL-2. It can inhibit the MAPKs-activated activator protein (AP-1) transcription factor in prostate cancer cells, eventually suppressing tumour growth [35,36]. It has been demonstrated to significantly reduce the levels of c-Jun N-terminal kinase (JNK) and of the epigenetic marker H3K4 in lymph node carcinoma of the prostate (LNCaP) cells [37]. In both androgen-dependent and androgen-independent prostate cancer cells, curcumin induces apoptosis by downregulating apoptosis suppressor proteins [38]. It has also been shown to block NF-κB activation and enhance TRAIL-induced cytotoxicity in LNCaP cells [39].

### 3.4. Brain Cancer

Brain tumours are very resistant to many kinds of therapy [40]. Nearly half of all brain tumours are classified as glioblastoma (GBM) [41,42]. Several studies have been conducted to enhance the delivery of curcumin through the BBB using nanoparticles, as curcumin, in its free form, has low permeability across the BBB [43]. Curcumin has been reported to exert an antiproliferative effect on GBM cells, significantly reducing the levels of non-coding RNAs (miR-21 and miR-378), which play a significant role in the progression of GBM. This reduction in the proliferation of the GBM stem cells by curcumin occurs via activation of the MAPK pathway and inhibition of the inhibitor of apoptosis (IAP) and STAT3-dependent pathways [44]. In many in-vitro studies, curcumin was reported to suppress the proliferation of GBM cells, controlling the expression levels of EGFR, linked to pathways such as the PI3K/Akt and the Janus kinase (JAK)/STAT-dependent pathways [45,46]. Curcumin administered with tyrphostin AG1478 (a type of EGFR kinase inhibitor) causes irreparable damage in DNA, decreasing the viability of GBM cancer cells [47].

### 3.5. Pancreatic Cancer

The occurrence of pancreatic cancer worldwide is low (3% of all cancers). This type of cancer, with a high level of metastasis, is very difficult to treat and has a high fatality rate [48]. Curcumin has been reported to exert antiproliferative activity on pancreatic stellate cells (PSCs), via suppressing platelet-derived growth factors and the phosphorylation of extracellular signal-related kinases [49]. Recent studies showed that curcumin, together with one of its synthetic derivatives, effectively suppresses tumours by acting on cancer stem cells (CSC) which are the root cause of tumour generation and proliferation [50,51]. Curcumin induces apoptosis in pancreatic cancer cells through the induction of forkhead box O1 and inhibition of the PI3 K/Akt pathway in PANC-1 cancer cells [52]. It downregulates the expression of the key oncogenic factor cell division cycle 20 (cdc20) protein. It increases the expression of p21 and Bcl-2-like protein 11 (Bim), reducing the motility of cancer cells and increasing apoptosis [52,53]. It also shows antiproliferative activity on PANC-1 cancer cells via decreasing the mRNA expression of the IPA protein [54] 

### 3.6. Gastric Cancer

Gastric cancer is the world’s third-most lethal cancer [55]. Similarly to other cancers, it is linked to several genes, molecular pathways, signalling molecules, and epigenetic patterns [56]. Curcumin exerts its effect on gastric cancers via inactivation of a number of signalling pathways such as extracellular signal-Regulated Kinases (ERK), Akt, Ras, PI3K, p53, Wnt-β, and MAPKs. Curcumin also inactivates the NF-κB signalling pathway, reducing the levels of inflammatory mediators including tumour necrosis factor (TNF)-α and various other chemokines and interleukins [57,58]. It has been reported to inhibit the growth of hepatic stellate cells (HSC), promoting p53 gene expression and causing apoptosis [59,60]. It also inhibits the proliferation of BGC-823 and SGC-7901 gastric cancer cells, via interaction with the P13K pathway [60]. Its antiproliferative effect on MKN45, SGC7901, and NCI N87 cells is via regulating Bcl-2 signalling and caspase pathways and inactivating the Wnt3 a/β-catenin/epithelial-mesenchymal transition (EMT) pathway [61]. 

### 3.7. Leukaemia 

Leukaemia represents 8% of all cancers worldwide. representing 30% of all cancer occurring in children [62]. Leukaemia can be classified into four subtypes, i.e., acute myeloid leukaemia (AML), acute lymphoblastic leukaemia (ALL), chronic myeloid leukaemia (CML), and chronic lymphocytic leukaemia (CLL) [63]. The aetiology of CML is directly linked to the expression levels of the P210 BCR-ABL protein translated by the breakpoint cluster region-Abelson (BCR-ABL) gene. This protein is involved in the progenesis of cancerous cells due to its association with different pathways such as MAPK, Ras, and Raf [64]. Curcumin inhibits the MAPK pathway by downregulating p210 BCR–ABL [65]. This downregulation, along with that of the heat shock protein 90 (Hsp90), increases the therapeutic effect of imatinib [65]. This downregulation, along with that of the heat shock protein 90 (Hsp90), increases the therapeutic effect of imatinib [64,65]. Curcumin inactivates NF-κB in KCL-22 myeloid cells, leading to apoptosis. It also upregulates the TNFα-related apoptosis-inducing ligand (TRAIL) in the same cell line [66]. When administered in combination with another plant polyphenol called carnosic acid, it induced a synergistic effect, inducing apoptosis in AML cells [67]. When administered in combination with daunorubicin, it increases the cytotoxicity of daunorubicin in CD34+ AML cells [68]. In AML cells, curcumin has been reported to decrease the levels of STAT5A and FLT3—a biomarker present in AML [69]. Among all hematological cancers, CLL is most common in the western world [70]. In this type of leukaemia, the levels of T cells and natural killer (NK) cells are high, and there is the presence of defective neoplastic B lymphocytes [71]. Curcumin has been reported to target the pathways related to the persistence of neoplastic B lymphocytes. It can downregulate the expression of Mcl-1, an X-linked inhibitor of apoptosis protein (XIAP), and inhibit the AKT, NF-κB, and STAT3-dependent pathways in vitro. It also leads to cleavage of the poly [ADP-ribose] polymerase-1 (PARP1)-dependent pathways. Curcumin targets various other signalling pathways associated with the progenesis of tumours (e.g., MEK/Raf/ERK and mTOR/Akt, STAT5) [72,73,74,75].

## 4. Clinical Trials of Curcumin in Cancer

The potential therapeutic effects of curcumin on cancer continue to draw great interest from the scientific community. There have been a number of clinical studies conducted on human subjects to evaluate the effectiveness and safety of treatment with curcumin, either alone or in combination with other drugs, and in various cancer types. A summary of the clinical trials conducted to date is presented in Table 5 and Table 6. So far, most clinical trials have explored the bioavailability of curcumin, how it affects distinct cancer types, and how well it works to mitigate the adverse effects of radiotherapy and chemotherapy. The results of these trials indicate that curcumin has a promising potential in the treatment of cancer. However, it is important to point out that research on the long-term usage of curcumin supplementation is still lacking, making it difficult to predict if this would elicit any chronic adverse effects. Prospective clinical studies ought to investigate the efficacy and bioavailability of various dosages and/or formulations of curcumin as well as confirm its synergistic effects with currently available cancer chemotherapeutics. Results from the current and upcoming clinical trials will provide a strong scientific basis for the clinical use of curcumin in cancer therapy.

## 5. Concluding Remarks and Future Perspectives

The activity of curcumin on different types of cancer, including breast cancer, chronic myeloid leukaemia, head and neck squamous cell carcinoma, colorectal cancer, prostate cancer, intestinal adenomas, and cervical cancer, has been demonstrated in numerous in vitro, in vivo and clinical studies. This effect is mediated via various pathways, including PI3K/Akt, JAK/STAT, MAPK, Wnt/β-catenin, p53, NF-κB, and apoptosis-related cell signalling. Curcumin has so far shown a promising role in cancer chemoprevention and chemotherapy. Future research is warranted to identify the most suitable formulation/dosage to be used to guarantee optimal concentrations of curcumin in the blood and tissues and achieve the best outcome. 

## Figures and Tables

**Table 1:** Natural curcuminoids and analogues of curcumin [1].

Compound Name	Plant Origin	Molecular Formula	Pubchem ID
Curcumin **(a)**	*Curcuma longa* (Turmeric)	C_21_H_20_O_6_	969516
Cyclocurcumin **(b)**	*Curcuma longa* (Turmeric)	C_21_H_20_O_6_	69879809
Bisdemethoxycurcumin **(c)**	*Curcuma longa* (Turmeric)	C_19_H_16_O_4_	5315472
Demethoxycurcumin **(d)**	*Curcuma longa* (Turmeric)	C_20_H_18_O_5_	5469424
Cassumunin A **(e)**	*Zingiber cassumunar* (Ginger)	C_33_H_34_O_8_	10460395
Cassumunin B **(f)**	*Zingiber cassumunar* (Ginger)	C_34_H_36_O_9_	10054109
Dehydrozingerone **(g)**	*Zingiber officinale Roscoe* (Ginger)	C_11_H_12_O_3_	5354238
6-Paradol **(h)**	*Zingiber officinale Roscoe* (Ginger)	C_17_H_26_O_3_	94378
6-Shogaol **(i)**	*Zingiber officinale* (Ginger)	C_17_H_24_O_3_	5281794
6-Gingerol **(j)**	*Zingiber officinale Roscoe* (Ginger)	C_17_H_26_O_4_	442793

**Table 2:** Physicochemical properties of curcumin [1,2].

Formula	C_21_H_20_O_6_
Chemical name	[(1*E*,6*E*)-1,7-bis(4-hydroxy-3-methoxyphenyl)hepta-1,6-diene-3,5-dione]
Molecular weight	368.38 g/mol
pKa values	First (pKa 7.7–8.5) Second (pKa 8.5–10.4) Third (pKa 9.5–10.7)
Stable at pH	Between 1–6
Num. heavy atoms	27
Num. rotatable bonds	8
Num. H-bond acceptors	6
Num. H-bond donors	2
Molar refractivity	102.80
Melting temperature	176 °C to 183 °C
Water solubility	0.4 mg/mL
Bioavailability score	0.55
Gastrointestinal absorption	High
Blood–brain barrier (BBB) permeant	No

**Table 3:** Approaches used to increase the bioavailability of curcumin [7,8,9].

Formulations	Curcumin Dose Administered	Plasma Levels of Curcumin
Use of lipid particles	650 mg	22.4 ng/mL at 2.4 h
	From 2 to 4 g	30–40 ng/mL between 2 to 4 h
Use of micelles	500 mg	1189 ng/mL at 1.1 h
	210 mg/day per 4 days	253 ng/mL (total curcuminoids)
Use of piperine	2 g + 5 mg	6.92 ng/mL (mean)
	4 g + 24 mg	136–176 ng/mL (range)
	2 g/kg + 20 mg/kg	180 ng/mL at 0.75 h
Use of hydrophilic nanoparticles	30 mg	1.8 ± 2.8 ng/mL
	376 mg	27.3 ± 6.4 ng/mL at 1.4 h
	30 mg	25.5 ± 12.2 ng/mL
	Multiple doses of 200 or 400 mg/day	324 ng/mL with a dose of 200 mg of Theracurmin^®^ and 440 ng/mL with a dose of 400 mg
	150 or 210 mg	189 ± 48 ng/mL with a dose of 150 mg and 275 ± 7 ng/mL with a dose of 210 mg

**Table 4:** Effects of curcumin on cell signalling pathways in different types of cancer.

Type of Cancer	Cell Signalling Pathway	Effect	Model Used	Dose Administered	References
**Lung Cancer**	Wnt/β-catenin	Downregulation/inhibition	Human cell line A549	60 µM	[10,11,12,13,14,15,16,17,18,19]
	VEGF	Downregulation/inhibition	Nude mice	100 mg/kg	
	NF-κB	Downregulation/inhibition	Nude mice	100 mg/kg	
	Notch 1	Downregulation/inhibition	Human lung cancer cell lines	6 µM	
	ERK 1/2	Downregulation/inhibition	Human NCI-H1975 line	10 ng/mL	
**Breast Cancer**	Akt/mTOR	Downregulation/inhibition	Human breast cell lines	10 or 30 µM	[20,21,22,23,24,25,26,27,28]
	NF-κB	Downregulation/inhibition	Human breast cell lines	20 or 25 µM	
	MDR-1	Downregulation/inhibition	MCF-7 breast cancer cell line	1.3 µM	
	Bcl-2 and Bcl- xL	Downregulation/inhibition	T47D human breast cells	20 µM	
	FEN1	Downregulation/inhibition	MCF-7 breast cancer cell line	0–50 µM	
	Autocrine GH	Downregulation/inhibition	T47D human breast cells	20 µM	
**Prostate Cancer**	Androgen receptor-dependent	Downregulation/inhibition	LNCaP cell line	0.25 µM and 0.5 µM	[29,30,31,32,33,34,35,36,37,38,39]
**Brain Cancer**	STAT3	Downregulation/inhibition	Human GBM stem cells	25 µM	[40,41,42,43,44,45,46,47]
	IAP	Downregulation/inhibition	Human GBM stem cells	25 µM	
	MAPK	Upregulation/activation	Human GBM stem cells	25 µM	
**Pancreatic cancer**	Platelet-derived growth factor	Downregulation/inhibition	Rat pancreatic stellate cells	25 µM	[48,49,50,51,52,53,54]
	PI3 K/Akt	Downregulation/inhibition	Panc-1 human pancreatic cells	20 µM	
	Cdc20	Downregulation/inhibition	Patu8988 and Panc-1 human cell lines	10 or 20 µM	
	IAP	Downregulation/inhibition	PANC-1 human cells	10/50/100 µM	
**Gastric cancer**	PI3K	Downregulation/inhibition	Human SGC-7901 and BGC-823 cells	10/20/40 µM	[55,56,57,58,59,60,61]
	BCL-2	Downregulation/inhibition	Human gastric cell lines	20 µM	
	Wnt3 a/β-catenin/EMT	Downregulation/inhibition	Human gastric cell lines	20 µM	
**Leukaemia-CML**	MAPK	Downregulation/inhibition	Human K562 cell line	5 or 10 mg/L	[62,63,64,65,66,67,68,69,70,71,72,73,74,75]
	p210 BCR-ABL	Downregulation/inhibition	Human K562 cell line	5 or 10 mg/L	
	Hsp90	Downregulation/inhibition	Human K562 cell line	30 µM	
**Leukaemia-CLL**	AKT	Downregulation/inhibition	Human CLL B cells	10–12.5 µM	
	NF-κB	Downregulation/inhibition	Human CLL B cells	10–12.5 µM	
	STAT3	Downregulation/inhibition	Human CLL B cells	10–12.5 µM	
	XIAP	Downregulation/inhibition	Human CLL B cells	10–12.5 µM	
	Mcl-1	Downregulation/inhibition	Human CLL B cells	10–12.5 µM	
**Leukaemia-AML**	MMP	Downregulation/inhibition	Human SHI-1 cells	6.25–25 µM	
	Bcl-2	Downregulation/inhibition	Primary human CD34+ AML cells	0–80 µM	
	MAPK	Downregulation/inhibition	Human SHI-1 cells	6.25–25 µM	
**Leukaemia-ALL**	AKT/mTOR	Downregulation/inhibition	Human ALL cell lines	0–40 µM	
	BCR/ABL	Downregulation/inhibition	Human ALL cell lines	0–40 µM	
	ABL/STAT5	Downregulation/inhibition	Human ALL cell lines	0–40 µM	

**Table 5:** Clinical studies on the effect of curcumin on different types of cancer.

Cancer Type	Study Type	Number of Patients in the Study	Treatment	Endpoints	Results	References
**Breast cancer**	Clinical trial	14	Docetaxel + Curcumin (0.5–8 g/day for 7 days)	VEGF and tumour markers levels; Maximal tolerated dose of curcumin; Efficacy; Safety; Toxicity	Decreased levels of VEGF; No cancer progression; Low frequency of toxic effects; Partial response in some patients	[76]
**Chronic Myeloid** **Leukaemia**	Randomized controlled trial	50	Imatinib (400 mg twice daily) + Curcumin (5 g three times daily for 6 weeks)	Plasma nitric oxide levels	Reduced nitric oxide levels	[77]
**Benign Prostatic** **Hypertrophy**	Pilot project	61	Curcumin (1 g per day for 24 weeks)	Quality of life; Signs and symptoms	Improved quality of life; Reduced signs and symptoms of the disease	[78]
**Head and Neck Squamous Cell Carcinoma**	Pilot study	21	Single dose of curcumin (1 g)	Cytokine levels and Iκkβ kinase activity in saliva	Reduced IκKβ activity in salivary cells	[79]
**Colorectal Cancer**	Dose-escalation pilot study	15	Curcumin (40–200 mg per day for 29 days)	PGE2 levels and COX-2 activity	Dose-dependent decrease in PGE2 levels	[80]
	Does-escalation trial (Phase I)	12	Curcumin (0.45 g, 1.8 g and 3.6 g per day for 7 days)	Concentrations of curcumin and its metabolites in plasma, and colorectal tissue	Concentrations of Biologically active curcumin in the colorectal tissue	[81]
	Does-escalation trial (Phase I)	15	Curcumin (0.45–3.6 g per day for 120 days)	PGE2 and glutathione S-transferase activity in blood; Concentration of curcumin and its metabolites in plasma, faeces and urine	Very low levels of curcumin and its metabolites in plasma and urine and dose-dependent decrease in PGE2 levels	[82]
	Pilot study	26	Curcumin (2.35 g per day for 14 days)	Tolerance, safety and levels of curcumin in the colonic mucosa	Prolonged biologically active levels of curcumin achieved in the colon. Safe and well tolerated	[83]
	Clinical trial (Phase I)	126	Curcumin (360 mg three times daily for 10–30 days)	p53 expression and TNF-α levels in serum and colorectal tissue	Increased expression of p53; Decreased levels of TNF-α in serum and tissue	[84]
	Clinical trial (Phase II)	44	Curcumin (2 and 4 g per day for 30 days)	Total number and concentration of 5-hydroxyeicosatetraenoic acid and PGE2 within aberrant crypt foci and normal mucosa	Reduced number of aberrant crypt foci with a dose of 4 g per day	[85]
**Prostate cancer**	Randomized controlled trial	85	Soy isoflavones (40 mg) + curcumin (100 mg) for 180 days	Prostate-specific antigen levels in serum	Decreased levels of prostate-specific antigen	[86]
	Randomized controlled trial	40	Radiotherapy + curcumin (3 g per day for 90 days)	Altered activity of antioxidant enzymes and biochemical and clinical progression-free survivals	Decreased levels of prostate-specific antigen and considerable antioxidant effect	[87]
	Clinical trial (Phase I)	16	Curcumin (200–400 mg per day for 270 days)	Safety, cytokine levels, pharmacokinetics, NF-κB activity, efficacy and quality of life	No noteworthy changes in NF-κB activity or cytokine levels, safe, good pharmacokinetics and improved quality of life	[88]
**Pancreatic cancer**	Clinical trial (Phase I/II)	21	Gemcitabine + curcumin (8 g per day for 14 days)	Efficacy, patient compliance and toxicity	Median overall survival time of 161 days; Safe and well tolerated	[89]
	Clinical trial (Phase II)	17	Curcumin (8 g per day for 30 days)	Toxicity profile and time to tumour progression	Tumour progression of 1–12 months and high frequency of side effects	[90]
	Clinical trial (Phase II)	25	Curcumin (8 g per day for 60 days)	Tumour markers, tumour response, and adverse effects	Biological response in only 2 patients, poor oral bioavailability, and no toxicity	[91]
**Intestinal** **Adenoma**	Randomized controlled trial	44	Curcumin (1.5 g twice a day for 12 months)	Mean polyp size, total number of polyps and adverse effects	No significant clinical response Very few adverse effects	[92]

**Table 6:** Recent clinical trials investigating the effect of curcumin on different cancer types [10].

Cancer Type	Treatment	Project Title	NCT *	Phase	Estimated/Actual Completion Date
**Breast Cancer**	Curcumin ^®^ (CUC-01)+ paclitaxel	Curcumin in Combination with Chemotherapy in Advanced Breast Cancer	NCT03072992	2	30 June 2019
**Colorectal Cancer**	Avastin/FOLFIRI + curcumin	Avastin/FOLFIRI in Combination with Curcumin in Colorectal Cancer Patients with Unresectable Metastasis	NCT02439385	2	1 August 2019
**Prostate Cancer**	Curcumin + radiation	Nanocurcumin for Prostate Cancer Patients Undergoing Radiotherapy (RT)	NCT02724618	2	April 2022
**Breast Cancer**	Curcumin	A ‘Window Trial” on Curcumin for Invasive Breast Cancer Primary Tumours	NCT03980509	1	30 December 2022
**Cervical Cancer**	Curcumin	Curcumin in Advanced Cervical Cancer	NCT04294836	2	31 December 2023
**Prostate Cancer**	Curcumin	Trial of Curcumin to Prevent Progression of Low-risk Prostate Cancer Under Active Surveillance	NCT03769766	3	November 2026

* NCT = National Clinical Trial.

## Data Availability

Not applicable.

## Abbreviations

ALL: Acute lymphoblastic leukaemia; AML: Acute myeloid leukaemia; BBB: Blood–brain barrier; Bcl: B Cell Leukemia; BCR-ABL: Breakpoint Cluster Region-Abelson; Bim: Bcl-2 Interacting Mediator; CLL: Chronic lymphocytic leukaemia; CML: Chronic myeloid leukaemia; CSC: Cancer stem cells; DNA: Deoxyribonucleic Acid; EGFR: Epidermal growth factor; ERK: Extracellular signal-regulated kinase; ERK: Extracellular signal-regulated kinases; EZH: Enhancer of zeste homolog; FEN: Flap Endonuclease; FLT: FMS-like tyrosine kinase 3; GBM: Glioblastoma; GH: Growth hormone; Her: Human epidermal growth factor receptor; HSC: Hepatic stellate cells; Hsp: Heat shock protein; IAP: Inhibitor of apoptosis; IPA: Ingenuity Pathways Analysis; JAK/STAT: Janus kinase/signal transducers and activators of transcription; JNK: c-Jun N-terminal kinases; LNCaP: Lymph node carcinoma of the prostate; MAPK: Mitogen-activated protein kinase; MCF-7: Michigan Cancer Foundation—7; MDR: Multidrug resistance mutation; mRNA: Messenger Ribonucleic Acid; NK Cell: natural killer cell; NSCLC: Non-small-cell lung cancer; PI3K: Phosphoinositide 3-kinase; PSC: Pancreatic stellate cells; ROS: Reactive oxygen species; SSAT: Spermidine/spermine N1-acetyltransferase; STAT: Signal transducer and activator of transcription; TNF: Tumour necrosis factor; TRAIL: TNFα-related apoptosis-inducing ligand; VEGF: Vascular endothelial growth factor; XIAP: X-linked inhibitor of apoptosis protein.
